# Effect of combined actions of hip adduction/abduction on the force generation and maintenance of pelvic floor muscles in healthy women

**DOI:** 10.1371/journal.pone.0177575

**Published:** 2017-05-24

**Authors:** Amanda C. Amorim, Licia P. Cacciari, Anice C. Passaro, Simone R. B. Silveira, Cesar F. Amorim, Jefferson F. Loss, Isabel C. N. Sacco

**Affiliations:** 1 Physical Therapy, Speech and Occupational Therapy, School of Medicine, University of Sao Paulo, São Paulo, Brazil; 2 University Hospital of the University of Sao Paulo, São Paulo, Brazil; 3 Physical Therapy Master Program, University of the City of Sao Paulo (UNICID), São Paulo, Brazil; 4 Federal University of Rio Grande do Sul, Porto Alegre, Brazil; University of Otago, XX

## Abstract

Pelvic floor muscle (PFM) force and coordination are related to urinary incontinence severity and to sexual satisfaction. Health professionals frequently combine classic PFM exercises with hip adduction/abduction contraction to treat these disorders, but the real benefits of this practice are still unknown. Based on a theoretical anatomy approach whereby the levator ani muscle is inserted into the obturator internus myofascia and in which force generated by hip movements should increase the contraction quality of PFMs, our aim was to investigate the effects of isometric hip adduction and abduction on PFM force generation. Twenty healthy, nulliparous women were evaluated using two strain-gauge dynamometers (one cylinder-like inside the vaginal cavity, and the other measuring hip adduction/abduction forces around both thighs) while performing three different tasks: (a) isolated PFM contraction; (b) PFM contraction combined with hip adduction (30% and 50% maximum hip force); and (c) PFM contraction combined with hip abduction (30% and 50% maximum hip force). Data were sampled at 100Hz and subtracted from the offset if existent. We calculated a gradient between the isolated PFM contraction and each hip condition (Δ Adduction and Δ Abduction) for all variables: Maximum force (N), instant of maximum-force occurrence (s), mean force in an 8-second window (N), and PFM force loss (N.s). We compared both conditions gradients in 30% and 50% by paired t-tests. All variables did not differ between hip conditions both in 30% and 50% of maximum hip force (p>.05). PFM contraction combined with isometric hip abduction did not increase vaginal force in healthy and nulliparous women compared to PFM contraction combined with isometric hip adduction. Therefore, so far, the use of hip adduction or abduction in PFM training and treatments are not justified for improving PFM strength and endurance.

## Introduction

Pelvic floor dysfunctions, such as pelvic organ prolapse and urinary and fecal incontinence, are conditions that are likely to affect over 60% of all women at some point in their lives [1].

The levator ani muscles play a well-defined role in urinary and fecal continence. When the levator ani activates, its resultant force, in a ventrocephalic direction, compresses the rectum, distal vagina, and urethra behind the pubic bone, resulting in an elevation of the urethrovesical neck. These muscles together with the ligaments are responsible for sustaining the pelvic organs and for maintaining the closure of the urogenital hiatus [2].

It has been shown that the levator ani present a delayed activity in women with urinary incontinence during coughing [3], a reduced passive force and endurance during sustained contraction, and a reduced rate of force development during rapidly repeated contractions in the same population [4]. Major structural abnormalities (avulsion and atrophy) of the levator ani (pubovisceral portion) were associated with pelvic organ prolapse in a prospective observational study [5]. All these functional and structural alterations related to urogynecological dysfunctions support the widespread recommendation that pelvic floor muscle (PFM) training should be the first-line conservative management for women with a variety of types of dysfunctions [6].

PFM training usually includes repeating contractions and relaxations of the PFM, commonly performed while isolated or in combination with contractions of the abdominal and hip muscles, such as gluteal and hip adductors and abductors muscles [7–9].

The result of such combination in the clinical practice is still a matter of debate in the literature and needs more evidence to support such practice. There are some published results that suggest this dynamic relationship between PFM and lower trunk/ hip region that could support the practice in PFM training. One study showed by intra-vaginal indwelling EMG, placed in the anterior wall of the urethra and lateral to the urethra in PFM, a coactivation among the striated urethral muscles, hip adductors, gluteal and abdominal muscles while performing some maneuvers in healthy subjects [10]. Other important studies also showed a coactivation between PFM and abdomen by surface EMG [11,12] and by ultrasound [13] during PFM contractions. In contrast, another study showed that only gluteal-muscle coactivation with PFM contraction truly contributed to the continence mechanism, resulting in bladder-neck elevation, but not when combined with hip-adductors contraction [14]. Therefore, there is still no consensus on the role of hip muscle activity on the pelvic floor mechanisms and function.

There is anatomical evidence suggesting that structures other than the levator ani and gluteal-muscles, especially the muscles surrounding the hip, such as the obturator internus, contribute to continence and to sexual function [15,16]. The obturator internus acts as the main hip abductor and external rotator, particularly with the hip flexed [17] and in isometric tasks [18]. Its covering fascia is attached to the iliococcygeus portion of the levator ani by the arcus tendineus fascia pelvis [15,16]. Therefore, despite the still-controversial scenario, it could be an opportunity for a different approach to PFM rehabilitation focusing on dynamic activation of the PFMs and the muscles surrounding the lower trunk and hip regions, particularly the muscles not yet well addressed.

Based on the intimate myofascial connection between obturator internus and levator ani, we proposed that this anatomical relationship creates a pivotal region of support, working in two ways. Firstly, we acknowledge that this myofascial connection may work as an anchoring point, for active and passive stabilization of pelvic organs and structures. Thus, active contraction of obturator internus can optimize levator ani dynamic action and force generation in the pelvic floor by providing a stronger anchoring area, supporting muscle optimal force and length. Secondly, it is possible that hip abduction increases tension in the levator ani through myofascial force transmission, summing tensions/forces in a serial arrangement, as shown previously [19,20], optimizing its action of squeezing and lifting the pelvic floor. As shown in finite elements models, additional force of one muscle comes from synergistic muscles via myofascial pathways [21]. Thus, better anchoring system for levator ani contraction and myofascial force transmission would then result in an increased anterior-superior force resultant in the vaginal canal, which ultimately would contribute to an efficient mechanical coordination.

We hypothesize that the combined action between PFM contraction and hip isometric abduction force may be beneficial for intra-vaginal force generation and maintenance, but not the hip adduction combined action, because of the inexistence anatomical and mechanical relationship between hip adductors muscles and PFM. It could represent a better choice for clinicians, particularly when compared to hip-adduction combinations done in clinical practice and protocol for PFM rehabilitation [8], when looking for strategies to improve proprioception and PFM recruitment, as well as facilitating rehabilitation. Considering intravaginal dynamometers as an ideal tool for precise measurements of PFM force and resistance [22,23], our aim was to investigate the effects of isometric force from hip adduction and abduction on PFM force performance in healthy continent women.

## Methods

### Participants

For this cross-sectional study, we recruited and evaluated twenty healthy, nulliparous women (27.2±5.3 years, 22.7±3.1 kg/m^2^ and 3.6±1.1 score on the Oxford scale) who were able to contract their PFMs correctly (≥ 2 according the 0–5 point Oxford grading system [24] were eligible for this assessment. The eligibility criteria also included nonvirgins or women who were not pregnant at the time of selection, aged 21–40, without major organ prolapse (PopQ < stage 2), as evaluated by the clinician [25], without urinary tract/vaginal infection or chronic/degenerative neurologic/musculoskeletal diseases or a history of any kind of urinary incontinence, or other diseases that may have interfered with pelvic floor function measurements. Adult women working or studying at University of Sao Paulo were invited by electronic media and by flyers at our department and School of Medicine. Then, eligibility criteria were checked and the selection of participants was made. All the subjects gave written consent to participate in the study, which followed the principles of the Declaration of Helsinki and was approved by the School of Medicine Ethics Committee (CAAE: 15959113.1.0000.0065).

### Instruments

**Fig 1:**
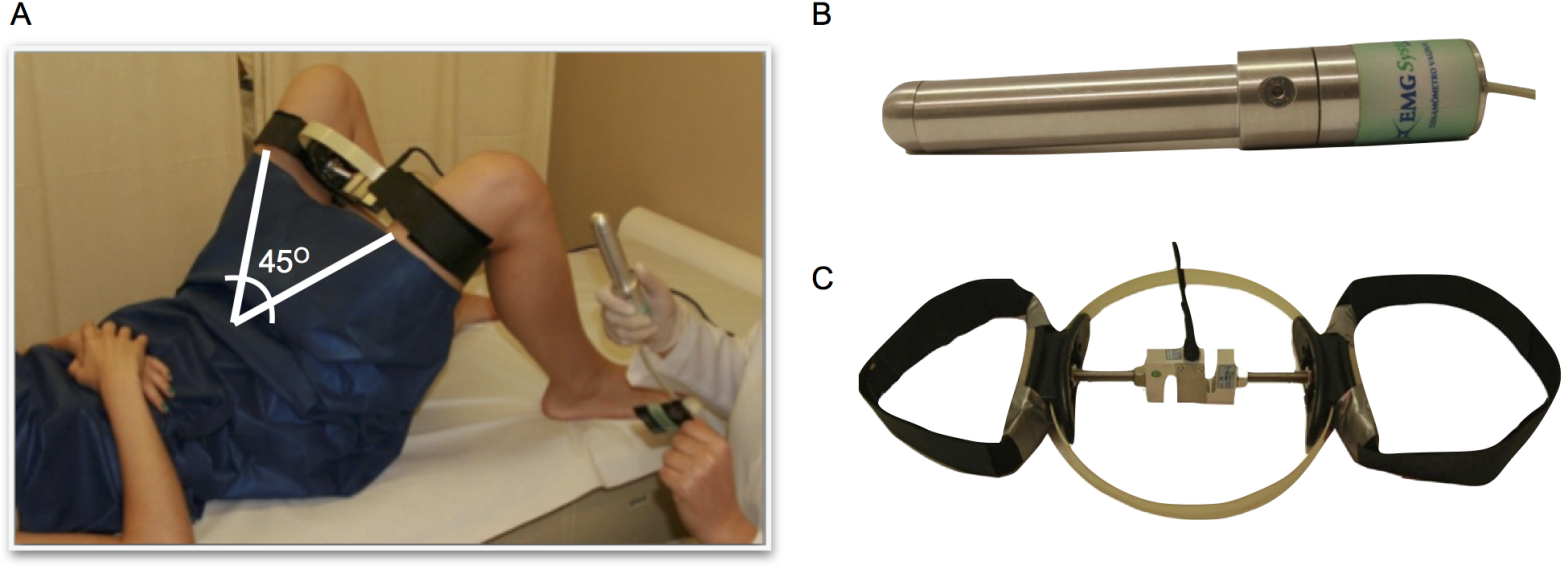
(a) Data acquisition set up, (b) intra-vaginal dynamometer, and (c) thigh dynamometer.

Two strain-gauge dynamometers were used simultaneously for intravaginal and hip adduction/abduction force measurement (Fig 1). The intravaginal dynamometer was designed for direct evaluation of PFM function (EMG-system do Brazil 386-2/2013, São José dos Campos, Sao Paulo, Brazil, amplifier gain 600, 0/2000N) and was instrumented with a strain-gauge at the distal part of the probe (in the position of the screw, Fig 1). A custom-made, ring-like dynamometer was built for measuring hip adduction and abduction force. The ring was 21 cm wide and was instrumented with an S-shaped load cell based on strain-gauge, bi-directional compression/tension, with a 0–100 kg range (EMG-system do Brazil 18AC66D/ 2013, São José dos Campos, Sao Paulo, Brazil). Transducer analog outputs were sampled at 100 Hz, synchronized, and converted to digital signals by a 16-bit A/D converter (National Instrument USB-6218 32 inputs, 250 kS/s, isolated multifunction I/O). The voltage values were converted into units of force (kgf) using a calibration factor: For the ring-like dynamometer, each volt corresponded to 38 kgf, and for the intravaginal dynamometer, each volt corresponded to 3.40 kgf.

### Clinical evaluation

The eligible participants were placed in a crook lying position on a conventional gynecologist’s table, based on Frawley et al. 2006 [26], in an isolated and comfortable private room. All assessments were performed in the same conditions.

The same physiotherapist (10 years’ experience with incontinent women) assessed PFM function by digital palpation with the two distal phalanges of the index and the middle fingers inserted into the vaginal introitus and positioned laterally [27]. The subjects were asked to squeeze and to lift their PFM as if preventing the escape of flatus and urine while breathing out. They received feedback from the physiotherapist about their PFM contraction during the preparation session [28]. The subjects who did not perform according to the instructions were excluded.

### Dynamometric evaluation of the pelvic floor and hips

Initially, we had to assess the maximum hip adduction and abduction isometric force for determining the 30% (or 50%) level that was used when the participants performed the PFM contraction with the vaginal probe inserted. For this determination, before inserting the vaginal dynamometer, the thigh dynamometer was positioned between the participant’s thighs, fixed by Velcro straps, and protected by a rubber support (Fig 1A). In this position, the participant kept her knees 21 cm apart, representing an aperture angle between the thighs of 45° (± 5), without any joint movement. The participant was then encouraged by the physiotherapist to perform maximum isometric force against the equipment and to sustain it until verbal feedback was provided. Three aperture trials of 3 seconds with a 1-minute rest period were recorded for each adduction and abduction condition, in a random order. The randomization process was performed by a Matlab simple code. A number was assigned for each task (1, 2, 3) for both 30 and 50% (A, B). The tasks and level of effort were then randomized for each one of the 20 subjects.

We calculated a mean of the three trials for each task, and, from these means, we determined a 30% (or 50%) level of the maximum hip adduction and abduction force. Those levels were determined in a pilot study during which we compared PFM forces while nine volunteers performed different levels of isometric hip force, together with PFM maximum contraction. In the 30% and 50% target levels, the subjects could perform both tasks simultaneously with a satisfactory level of PFM force.

After this procedure, the vaginal probe was covered with two condoms appropriately lubricated with hypoallergenic gel and inserted 7 cm deep from the hymeneal caruncle, always with the load cell facing up. To ensure correct positioning, a permanent pen was used to mark the 7 cm and right-probe direction on the first condom. After a 1-minute accommodation period, the participants were then asked to contract their PFMs as they did in the digital examination. Next, the participants were asked to perform maximum PFM contraction while (1) performing 30% (or 50%) of the maximal isometric hip adduction against the ring-like thigh dynamometer (adduction condition = adduction hip + PFM contraction), (2) 30% (or 50%) of the maximal isometric hip abduction against the thigh dynamometer (abduction condition = abduction hip + PFM contraction), and (3) isolated PFM contraction without performing any force against the thigh dynamometer (isolated condition = PFM contraction only).

**Fig 2:**
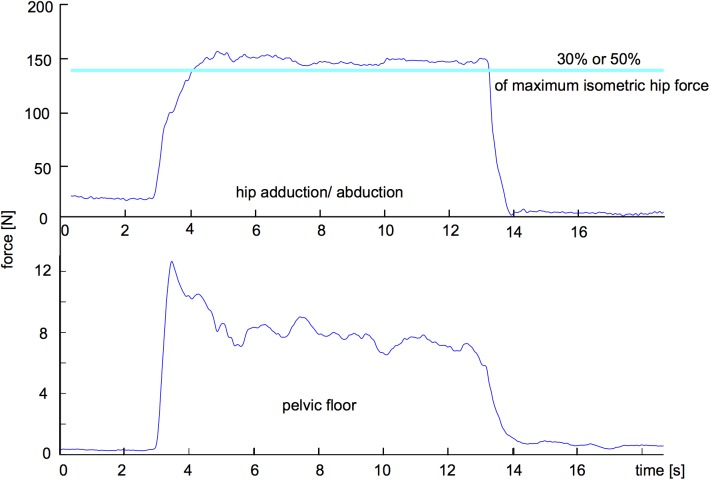
Example of visual feedback during data acquisition: time series of force data from the thigh dynamometer (above graph) and the target level of 30% of hip isometric force; time series of force data from the vaginal dynamometer (bellow graph).

Subjects received visual feedback of the hip force during the entire assessment to guide them in achieving the required percentage of their maximal isometric hip force (Fig 2). Standardized verbal encouragement was given throughout the effort [29]. The subjects were instructed not to hold their breath while performing PFM contraction (verbal command "breathe normally while contracting the muscles of your PF, do not hold your breath").

We recorded only those contractions resulting in an inward movement of the perineum [30] and occurring simultaneously with achievement of the target level of hip force, which was visually inspected on the screen (Fig 2). During each task (1, 2, and 3), we recorded three maximum PFM contraction trials of 10 seconds [31] with a 1-minute rest between them and in a random order. The subjects repeated this same protocol 4 weeks later for the 30 or 50% of hip maximum force, whose order was determined by randomization.

The choice of a 4-week period between measurements was preferred because it corresponds to a complete menstrual cycle [31]. Verbal feedback was given to the participants while the examiner visually inspected the PFM contraction to ensure the participants did not perform a Valsalva maneuver, and to ensure a correct squeeze and lift component. Cases where the dynamometer was thrown out, or did not show a proper movement direction (inward ascending), were excluded from the analysis.

### Data analysis

**Fig 3:**
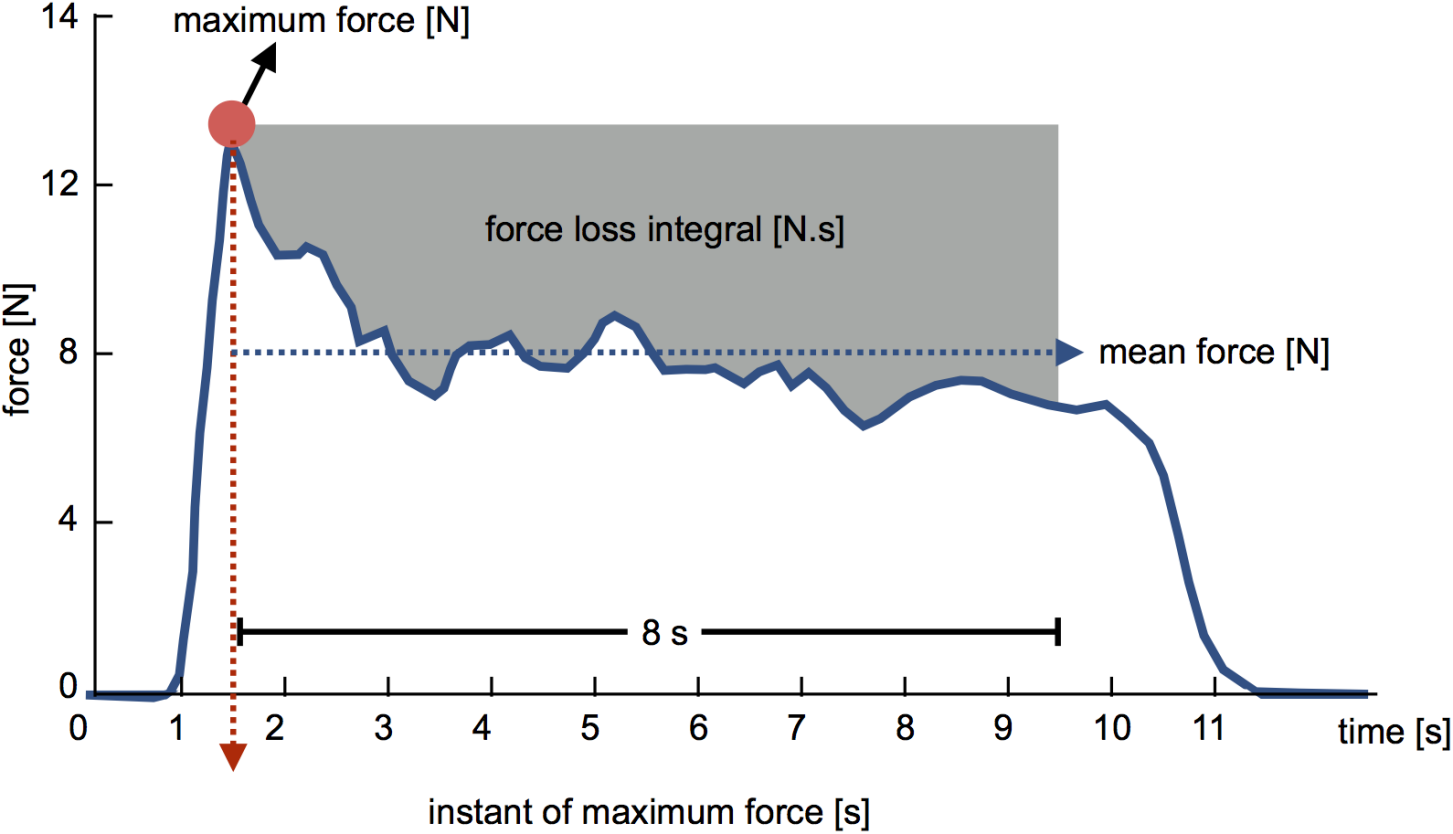
Variables calculated from dynamometric assessment of PFM contraction: PFM maximum force (N), mean force (N), instant of maximum force (s), and PFM force loss integral (N.s).

Vaginal dynamometer data were processed with a fourth-order, zero-lag, Butterworth low-pass filter with 8 Hz of cut-off frequency and subtracted from the offset if existent. Using a custom-written MATLAB function, we calculated the following variables: PFM maximum force (N); instant of maximum-force occurrence (s); mean force (from the first peak in an 8-s window) (N); PFM force loss, which is the difference between a rectangular area based on maximum force and the 8-second window (starting from the first peak force) and the area above the force-time curve at the same time (N.s) (Fig 3) for both 30 and 50% of maximum hip force. The mean of three trials was used for statistical purposes.

The pelvic floor isolated contraction is considered the gold standard for treatment of urogynecological dysfunction [7,9], therefore we calculated the supposed “gain” in adding hip abduction combined with the pelvic floor contraction, and the supposed “drawback” considering the hip adduction. We calculated a gradient to interpret the adduction and abduction tasks taking into account the isolated condition for all variables (Δ Adduction = isolated condition—adduction condition and Δ Abduction = isolated condition -abduction condition). We compared for all variables the Δ Adduction and Δ Abduction by paired t-tests (p<0.05). Given the sample size evaluated, a paired t-test as the statistical design, an alpha error of 5%, the statistical power (1- β) obtained was 0.882.

### Reliability of vaginal dynamometric assessment procedure

The test–retest reliability of the PFM force assessment was performed between trials and days (n = 11 women, 55% of total sample). The vaginal force assessment while performing the isolated PFM contraction task was repeated twice at 4-week intervals, by the same physiotherapist and under the same conditions and in the same environment described previously. The intraclass correlation coefficient (ICC) was calculated to determine reliability among the trials (3 trials–ICCt) (ICC3,1) and days (2 days–ICCd) (ICC3,k) [32]. The reliability between trials was excellent (ICC>0.75) for the PFM maximum force, mean force and PFM force loss and satisfactory (0.4≤ICC<0.75) for the instant of maximum force. The reliability between days was excellent (ICC>0.75) for all variables (Table 1).

**Table 1:** Intraclass correlation coefficients (ICC) between trials and days (ICCt and ICCd) of measurements calculated for the vaginal dynamometric variables.

Dynamometric variables (n = 11)	*ICCt 3*,*1*	*ICCd 3*,*K*
PFM maximum force (N)	0.95	0.98
Instant of maximum force (s)	0.68	0.89
Mean Force (N)	0.93	0.98
PFM force loss (N.s)	0.90	0.88

## Results

Demographic, anthropometric and clinical characteristics of the subjects are presented in Table 2. The comparisons of Δ adduction and Δ abduction revealed no statistical difference while performing a 30% maximum hip force for any of the analyzed variables: PFM maximum force (p = 0.73), instant of maximum-force occurrence (p = 0.73), mean force (p = 0.53) and PFM force loss (p = 0.57). There was also no statistical difference for 50% maximum hip force task: PFM maximum force (p = 0.07), instant of maximum-force occurrence (p = 0.37), mean force (p = 0.25) and PFM force loss (p = 0.11) (Table 3).

**Table 2:** Demographic characteristics of all subjects. Mean (standard deviation) of age and BMI, and number of subjects in each Power score (P)*.

	**Age (years)**	BMI** **(kg/m^2^)**	**P score 2**	**P score 3**	**P score 4**	**P score 5**
N = 20	28.5±5.3	21.9±3.2	1	13	6	-

*Modified Oxford grading (27)

**BMI: Body Mass Index.

**Table 3:** Mean (standard deviation), confidence interval [95%] and effect size (confidence interval of effect size) of PFM maximum force (N), instant of maximum force (s), mean force (N.s) and force loss integral (N.s) from all conditions (hip adduction, abduction and isolated pelvic floor contraction) and the calculated gradients.

**Variables**	**% of MVC**	**Adduction**	**Abduction**	**Isolated**	**Δ Adduction**	**Δ Abduction**	***t; p***	**Effect Size (CI of Effect Size)**
**Maximum force (N)**	30%	8.31±3.57 [6.59;10.03]	8.12±3.68 [6.35;9.90]	9.63±4.32 [7.55;11.72]	1.51±2.55 [1.92;3.77]	1,33±1.97 [1.48;2.91]	-0.35; 0.72	0.08 [-0.61;0.77]
	50%	7.80±4.6 [5.89;9.78]	7.20±3.60 [5.69;8.73]	10.30±6.50 [7.56;13.07]	3.38±3.39 [2.63;4.75]	2.70±2.66 [2.07;3.73]	-91.28; 0.07	0.23 [-0.69;1.15]
**Instant of maximum force (s)**	30%	3.52±1.61 [2.74;429]	3.42±1.19 [2.84;3.99]	2.32±1.82 [1.44;3.19]	1.10±2.41 [1.81;3.55]	1.20±2.19 [1.65;3.23]	0.34; 0.73	0.05 [-0.74;0.6]
	50%	4.90±3.10 [3.60;6.21]	4.30±2.40 [3.30;5.32]	2.30±2.60 [1.26;3.42]	1.97±3.50 [2.72;4.91]	2.56±3.30 [2.56;4.63]	0.90; 0.37	0.18 [-1.21; 0.85]
**Mean force (N)**	30%	6.07±3.12 [4.57;7.58]	5.80±2.85 [4.43;7.18]	8.00±3.98 [6.08;9.92]	2.20±2.52 [1.90;3.72]	1.93±2.26 [1.70;3.33]	-0.63; 0.53	0.11 [-0.61;0.84]
	50%	5.40±3.60 [3.88;6.89]	5.00±3.10 [3.74;6.36]	8.10±5.30 [5.84;10.32]	3.0±2.9 [2.24;4.04]	2.7±2.7 [2.12;3.83]	-16.63; 0.25	0.11 [-074;0.96]
**Force loss integral (N.s)**	30%	17.64±7.69 [13.93;21.34]	18.43±7.95 [14.59;22.26]	13.25±4.79 [10.94;15.56]	5.18±6.97 [5.26;10.30]	4.39±7.20 [5.44;10.65]	-0.56; 0.57	0.11 [-2.03; 2.26]
	50%	18.80±9.50 [14.77;22.81]	17.0±9.40 [13.06;21.01]	21.50±14.30 [15.42;27.52]	-5.40±12.19 [9.47;17.09]	-3.40±11.57 [8.99;16.22]	1.63; 0.11	0.17 [-3.42; 3.76]

## Discussion

Our main results do not support our hypothesis, which states that PFM contraction combined with hip isometric abduction spontaneously benefits intra-vaginal force production and maintenance, when compared to the hip adduction combined action. This hypothesis was based on our anatomical premise that there is epimuscular myofascial force transmission of the obturator internus to the levator ani during abduction and lateral hip rotation and also by providing a stronger anchoring area, would optimize PFM function. The simultaneous contraction of the PFM and the hip abductors or adductors did not improve pelvic floor efficiency with respect to increasing or holding its strength for the period evaluated. Therefore, we can conclude that the immediate effect of combining PFM contraction with hip isometric abduction or adduction does not have the claimed benefit for sustained PFM contraction practices.

Although Bø and Stien (1994) [10] showed a coactivation between hip adduction and PFM using indwelling EMG, our results did not reveal that hip adduction (or abduction) improve PFM force production and maintenance. Even though there was reported co-contraction between these two muscle groups, Peschers et al. (2001) [14] showed that only the gluteal-muscle coactivation with PFM contraction contributed to the continence mechanism, but not when combined with hip-adductors contraction. In our study we measured intra-vaginal forces directly using a dynamometer, and the contrast in the results from Bo and Stien’s study and ours may be related to different type of assessment. Indwelling EMG signal is hardly related to force especially in dynamic conditions such as evaluated here, and we showed that the association with the adduction task did not represent any augmentation in force parameters.

There is scientific evidence showing that force can be transmitted across compartmental barriers and between muscles and their surroundings through epimuscular myofascial pathways, changing serial sarcomere length heterogeneity and then, additional force can be added to one muscle from other muscles [19,33]. The pubovisceral and puborectal portions act mainly as a supportive hammock under the urethra and vesical neck, compressing the rectum, vagina, and urethra against the pubic bone during increases of intra-abdominal pressure (dynamic portion) [34]. The iliococcygeous portion of the levator ani, which functions as a flat support layer between both pelvic sidewalls, has a primary role of supporting the pelvic organs. It attaches the connective tissue of the arcus tendineous fascia pelvis to the obturator internus, which defines the pelvic (levator ani) and hip (obturator internus) compartments [34]. In this case, it is possible that the connective tissue of the arcus tendineous fascia pelvis not only serves as an anchor support for the PFM but also functions as a force transmitter between hip and pelvis. Unlike the hip abductors, and especially the obturator internus, the hip adductor muscles originate in the pubis and ischial bone, and their insertion occurs mainly in the posterior medial femoral surface, without any morphological relationship with any PFM. These are the main reasons why we hypothesized that the joint action between hip abductors—and not adductors—would lead to a greater force generation and maintenance then isolated contraction of the PFMs. However, within the context of this study design, we were unable to prove this theory.

Although we would expect differences between isometric hip abductor and hip adductor contractions in PFM force production, we have also to take into consideration the nature of the measuring tool that we used when interpreting our results. The dynamometer could not be sensitive enough to detect force increments when abducting or adducting the hips. Assessing the PF muscles’ physical capacities is still a matter of debate in the literature [35–37], mainly because of the complex pelvic floor structure with multiple origins and insertions [34]. Developing a quantitative 3-D tool to discriminately assess forces distributed through various regions of the vaginal canal and PFM layers and functions is still ongoing [38], and might have helped to discriminate the force increment while performing different combined tasks.

Another issue that could interfere in the results was the type of feedback delivered in each condition assessed. To assure that the right amount of hip force was accomplished and sustained (10 seconds), the volunteers were instructed to follow the visual augmented feedback of the load cells of the hip adductor/abductors. For maximum contraction of the pelvic floor, the volunteers had only augmented audio feedback from the evaluator. To avoid different influences of the type of augmented feedback in force performed, we used for comparisons the difference in force of each hip condition (adduction/ abduction) from the isolated task, therefore we ensure that both performances were obtained in equal conditions of feedback: adduction and abduction.

It is common to include in PFM training exercises consisting of isolated PFM contraction or in combination with contractions of the abdominal and hip muscles, such as gluteal and hip adductors muscles. However, the acute practice of combining hip adduction or abduction was not proven to be based in biomechanical evidences.

To ensure that the amount of hip force performed was adequate to verify a positive effect of pelvic floor exercises combined with hip abduction and adduction during clinical practice, we tested two submaximal hip contractions: 30 and 50%. In low levels of contraction (30%), it may be more complex and thus more difficult to control hip isometric force, because the subject must control motor unit firing, synchronization, and switch to achieve the specific target of the force task; therefore, the participants were also tested in higher level of contraction. But, even though they kept the same result as in low levels, not showing differences between adduct and abduct the hips together with PFM contraction. Our results give us confidence to conclude that this finding was not due to the amount of force asked to be performed.

PFM training is recommended for most urogynecological disorders [9]; thus, new and efficient strategies should be developed to improve the efficiency of this treatment. However, our results suggest that there is no evidence to include hip adduction or hip abduction for achieving PFM maximal force. It is worthy to note that the chronic effect of contractions combining PFM and hip abductors in a clinical context may achieve different results from those we observed in an immediate-effect evaluation. Training women in the conditions of combined tasks may improve the force generation and maintenance of the pelvic floor, since urinary loss frequently occurs while women perform daily living activities of a dual-task nature, such as walking, coughing, and lifting weights. However, further clinical trials are necessary to confirm this hypothesis.

Deeper understanding of the boundaries of PFM structures and connective tissues (pelvic and endopelvic myofascial structures), muscle fibers composition and neuromuscular control are mandatory for a better diagnosis and treatment of urogynecological dysfunctions. However, its intricate muscles layers organization, joined with connective tissues, complicates an accurate measurement, within an acceptable error limit, with the technological tools available today, such as dynamometers, perineometers and EMG. Developing a reliable, multidimensional mechanical tool capable of measuring the magnitude of loads, their temporal and spatial distribution, and the relative direction of PFM contractions would contribute to better understanding the potential trainability and capacity of the PFM in the pursuit of better sexual and continence functioning.

We evaluated healthy, nulliparous women without pelvic complaints and with healthy pelvic floors; perhaps, in cases of pelvic floor disorders such as urinary incontinence, PFM force generation and maintenance might be different, particularly in the combined conditions, and our initial hypothesis would be confirmed. A criticism of the present study might be that the evaluations were performed with young women, in whom urogynecological abnormalities are not frequent. Even so, our results could provide support to future studies dedicated to investigate if, in such cases, dysfunctional women would achieve different maximum forces would achieve different maximum forces during a joint action with the abductors that might compensate a probable PFM weakness.

## Conclusion

Isometric hip adduction or abduction combined with PFM contraction did not increase intra-vaginal force, which is associated with increasing levator ani force production, in healthy nulliparous women. Therefore, so far, the use of hip adduction or abduction in PFM training and treatments are not justified for improving PFM strength and endurance.
